# Loss of Ku70 promotes mononucleate conidiation and homologous recombination in *Phanerochaete chrysosporium*

**DOI:** 10.1128/aem.00497-26

**Published:** 2026-09-14

**Authors:** Hao Wu, Jiaojiao Zhang, Yi Zhao, Lan Yao, Aiqun Yu, Yi-Heng P. J. Zhang, Kuanqing Liu

**Affiliations:** 1 College of Biotechnology, Tianjin University of Science and Technology 66345, Tianjin, China; 2 In Vitro Synthetic Biology Center, Tianjin Institute of Industrial Biotechnology, Chinese Academy of Sciences 165087, Tianjin, China; 3 National Center of Technology Innovation For Synthetic Biology 658577 , Tianjin, China; 4 College of Food Science and Nutritional Engineering, China Agricultural University 34752, Beijing, China; 5 State Key Laboratory of Food Nutrition and Safety, Key Laboratory of Industrial Fermentation Microbiology of the Ministry of Education, Tianjin Key Laboratory of Industrial Microbiology, College of Biotechnology, Tianjin University of Science and Technology 66345, Tianjin, China; 6 State Key Laboratory of Engineering Biology for Low-Carbon Manufacturing, Tianjin, China; Royal Botanic Gardens Kew, Surrey, United Kingdom

**Keywords:** *Phanerochaete chrysosporium*, white-rot fungi, Ku70, transformation, homologous recombination, non-homologous end joining, mononucleate conidiation

## Abstract

Lignin is a major constituent of lignocellulose and the most abundant aromatic biopolymer on earth. It provides plants with rigidity and protection, but its recalcitrant nature also presents a significant barrier to lignocellulose valorization. The white-rot fungus *Phanerochaete chrysosporium* is among nature’s most efficient lignin degraders, and its ligninolytic capabilities have been subjected to intensive investigations. Genome editing with precision is crucial for elucidating the *in vivo* mechanisms of its ligninolytic actions, but genetic manipulations of *P. chrysosporium* are often plagued by imprecision. This technical nuisance is driven primarily by canonical non-homologous end joining (c-NHEJ), a DNA repair system that requires little homology and depends on the binding of the Ku70/Ku80 heterodimer to double-strand break (DSB) ends. Loss of Ku70 or Ku80 abolishes c-NHEJ and significantly improves genome editing precision in many filamentous fungi, but it has yet to be examined and exploited in *P. chrysosporium*. Here, we constructed a homozygous *ku70*Δ mutant in a meiotic homokaryon of clear genetic background. Loss of Ku70 minimally impacts growth but significantly increases homologous recombination frequency from ~2% to ~66%, with ~32% of the latter being homozygous. Unexpectedly, loss of Ku70 also promotes mononucleate conidiation, which may facilitate isolation of homozygous mutants. Taken together, our work provides a valuable genetic tool to understand and exploit *P. chrysosporium*’s remarkable ligninolytic capabilities.

**IMPORTANCE:**

Genome editing with precision is essential to unraveling the intricacies of *P. chrysosporium*’s exceptional ligninolytic capabilities, but the available tools are generally imprecise due to the dominance of non-homologous recombination, a problem that is further exacerbated by the discontinuation of Novozyme 234. We tackle these challenges by reestablishing protoplast-based transformation with Lywallzyme as an alternative. Importantly, we demonstrate that inactivation of c-NHEJ by deleting *ku70* significantly increases gene knockout efficiency and report the unexpected involvement of c-NHEJ in regulating the number of nuclei during conidiation. Our work paves the way for future ventures into understanding ligninolysis in *P. chrysosporium* and building superior chassis for industrial applications.

## INTRODUCTION

Lignocellulose is the major constituent of plant biomass and the largest renewable resource for chemicals and energy (1). Lignin, a major component of lignocellulose (the other two being cellulose and hemicellulose) and the second-most abundant biopolymer on earth, is a complex aromatic polymer synthesized via free-radical condensation of monolignols (2). Lignin endows terrestrial plants with rigidity and protection (3), but its recalcitrant nature (4) also poses a great challenge to lignocellulose valorization (5–9). Among the handful gifted lignin degraders in nature, a group of filamentous fungi collectively known as white-rot fungi (WRF), owing to their bleached appearance on lignocellulosic materials, truly stand out as they possess extraordinary capabilities to depolymerize and utilize lignin (10). Among them, the basidiomycete *P. chrysosporium* is the most studied and best understood WRF, and it can completely mineralize lignin-related compounds (LRCs) into carbon dioxide (11–15). Underlying this remarkable feat are an intricate group of enzymes featuring lignin peroxidases (LiPs) (16, 17), manganese peroxidases (MnPs) (18), auxiliary H_2_O_2_-generating oxidases (19–22), and enzymes involved in further metabolizing the resultant LRCs (23–28). Although these enzymes have been subjected to intensive biochemical characterizations, their *in vivo* contribution to lignin degradation and utilization has been underexplored. Furthermore, ligninolysis in *P. chrysosporium* is apparently a regulated process: it is a phenomenon during secondary metabolism, typically activated in response to starvation for nitrogen (29), carbon, or sulfur (30), often coinciding with the rise of cyclic AMP (cAMP) (31, 32). However, the underlying regulatory mechanisms remain poorly understood.

It is of great interest, from both basic and translational research perspectives, to understand the *in vivo* mechanisms of ligninolysis and its intricate regulations in *P. chrysosporium*. Such endeavors will undoubtedly benefit from genome editing tools with precision. In the late 1980s, the Gold group established a protoplast-based transformation protocol for *P. chrysosporium* via complementation of adenine auxotrophy (33), which was quickly adopted and further expanded to include G418 (34), phosphinothricin (PPT) (35), and pyrithiamine (36) as additional selection reagents. Moreover, DNA can also be delivered through electroporation (37) or *Agrobacterium tumefaciens* (38). Although powerful, these methods are generally marred with imprecision as the transformed DNA is often integrated into the genome via non-homologous recombination (33, 39), thus hampering genetic analysis, especially when well-controlled precision is desired, e.g., deleting a gene of interest to examine its importance in regulation and function of ligninolysis.

The aforementioned imprecision in genome editing is, however, not confined to *P. chrysosporium* but rather prevalent in filamentous fungi, animals, and plants. This technical nuisance is driven by non-homologous end joining (NHEJ), which operates as a crucial mechanism to repair DNA double-strand breaks (DSB) (40). Canonical NHEJ (c-NHEJ), the best understood and typically the dominant NHEJ pathway, requires little sequence homology and proceeds by binding of the Ku70/Ku80 heterodimer to DSB ends, which facilitates subsequent loading of DNA polymerase, nuclease, and ligase complexes to eventually join the DSB ends (41). Loss of either Ku70 or Ku80 is expected to abolish c-NHEJ, which would usher homologous recombination (HR) as a major pathway to mend DSBs, thus improving genome editing precision. Indeed, loss of Ku70 or Ku80 in *Neurospora crassa* drastically increased HR frequency (42), a phenomenon that has since been similarly observed and extensively exploited in many other filamentous fungi (43–46). However, the importance of Ku70 or Ku80 in HR has not been investigated in *P. chrysosporium*.

Here, we first isolated meiotic homokaryons of *P. chrysosporium* and performed whole-genome sequencing to clarify their genetic background. We then reestablished protoplast-based transformation with Lywallzyme and constructed a homozygous *ku70*Δ mutant. Loss of Ku70 drastically increased HR frequency at *mnp1* from ~2% to ~66%, and approximately one-third of the latter was homozygous *mnp1*Δ. Unexpectedly, we found that loss of Ku70 also promoted mononucleate conidiation, revealing for the first time the involvement of c-NHEJ in regulating the number of nuclei in conidia. Collectively, our work provides a valuable genetic tool for understanding *P. chrysosporium*’s remarkable ligninolytic capabilities and uncovers a previously unknown connection between c-NHEJ and mononucleate conidiation.

## RESULTS

### Isolation of meiotic homokaryons

Homokaryons are cells that contain only a single type of nucleus and are powerful tools for genetic analysis due to their presumed genetic homogeneity. *P. chrysosporium* RP-78, a popular homokaryon of non-meiotic origin, was constructed by the Cullen group in 2000 (47), whose genome was sequenced in 2004 (48). However, such a strain was not readily available to us; therefore, we decided to obtain homokaryons via basidiospores due to technical ease and simplicity. Meiotic homokaryons have been widely employed for genetic analysis in *P. chrysosporium* (49–52), although we were aware of potential meiotic recombination that might lead to genetic heterogeneity (50, 53–55). Nevertheless, we followed a method published by Gold and Cheng (56) (see Materials and Methods for details) to induce fruiting in BKMF-1767. Although basidiospores were readily obtained, the yield was orders of magnitude lower than that reported for another heterokaryon ME446 (56). The poor basidiospore yield in our work was similarly observed by James et al. (57), although their fruiting method differed from ours. Nonetheless, as we decided to employ these basidiospores for subsequent analysis, it was imperative to perform a more thorough characterization. To this end, we selected two isolates, named B1 and B3, and amplified the *lipI* gene, the size of which was previously used to distinguish between homokaryons and heterokaryons (47). As expected, amplification from the heterokaryotic BKMF-1767 showed two bands, a larger amplicon corresponding to the *Pce1*-interrupted *lipI* and smaller *Pce1*-free *lipI* (Fig. S1A). By comparison, both B1 and B3 showed the longer form (Fig. S1A), a feature also shared by RP-78, consistent with the notion that both isolates were homokaryons. We next sequenced their genomes and found that overall B1 and B3 were highly similar to the published RP-78 reference genome, although differences were apparent. Tentatively, 1,528 and 1,499 single-nucleotide polymorphisms (SNPs) and insertion/deletions (indels), as well as 31 and 37 structural variations (SVs), were identified in B1 and B3, respectively (Tables S1 and S2). We did not further scrutinize these changes; therefore, the actual alterations were expected to be fewer. Nevertheless, B1 and B3 were virtually indistinguishable from each other, and we could confidently identify only one SNP at 2412491 of Scaffold 6 in B1, which was absent from B3 and the parental BKMF-1767 (Table S3 and Fig. S1B and C). This change caused a T-to-C transition at the putative 3′ untranslated region (UTR) of gene_6426, but its presence did not appear to impact growth or conidiation (Table S3 and Fig. S1D and E). Taken together, these results indicate that B1 and B3 are homokaryons of highly similar genetic background, and both resemble RP-78.

### Reestablishment of protoplast-based transformation

**Fig 1:**
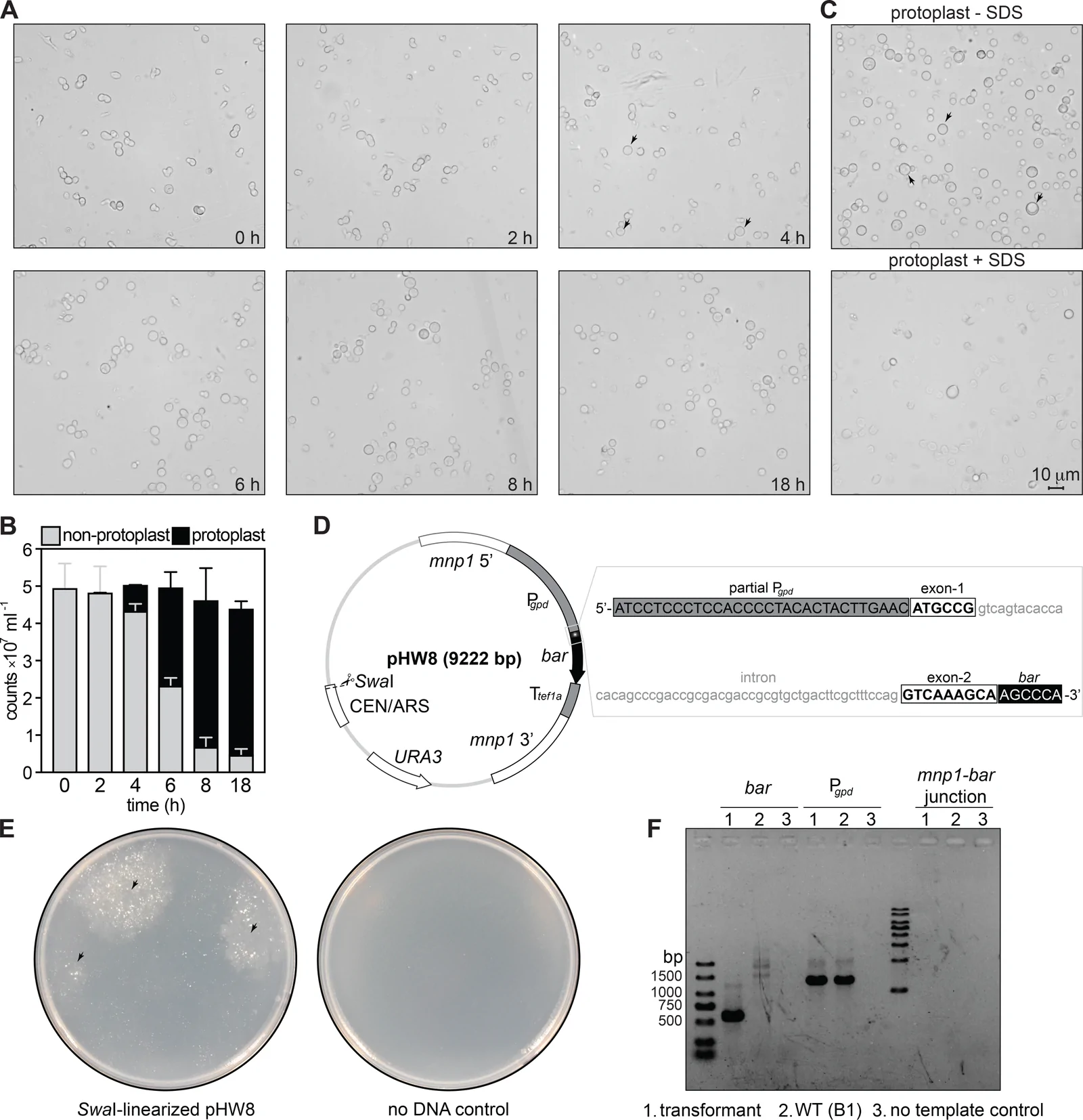
Reestablishment of protoplast-based transformation for *P. chrysosporium*. (**A**) Protoplasting WT (B1) conidia collected from PDA plate with Lywallzyme. (**B**) Time-dependent protoplasting efficiency. Mean ± s.d. (*n* = 3 biological replicates). (**C**) Protoplasts were extremely sensitive to SDS. An aliquot of 10 µL of protoplasts (18 h) was gently mixed with 30 µL of OM buffer or 30 µL of 10% SDS and examined under a light microscope. (**D**) Schematic of pHW8 used for transformation. The gradient box indicates the first two exons and the first intron of *gpd* (see the inset for details). (**E**) Appearance of PPT-resistant transformants (black arrows) on GK + 0.4 M MgSO_4_ + 200 µg mL^−1^ PPT with *Swa*I-linearized pHW8. (**F**) PCR diagnosis of a representative transformant showing that linearized pHW8 was integrated into the genome likely via non-homologous recombination.

Functional investigation of a gene of interest *in vivo* (e.g., *ku70* of the c-NHEJ pathway) requires a robust transformation protocol. Early transformation of *P. chrysosporium* primarily relied on generation of protoplasts using Novozyme 234 (33, 58), which was unfortunately discontinued. Although alternative DNA delivery methods using electroporation (37) or *A. tumefaciens* (38) have been reported, protoplast-based transformation remains a popular approach due to its simplicity. To find an alternative to Novozyme 234, we tested a domestic enzyme cocktail, branded Lywallzyme from the Institute of Microbiology, Guangdong Academy of Sciences. We replaced both Novozyme 234 and Cellulase CP with Lywallzyme in the original protoplasting protocol (33) and closely monitored protoplast formation under a light microscope. Protoplasts began to appear after 4 h and continued to accumulate until 8 h (Fig. 1A). Further incubation did not significantly increase protoplast yield (Fig. 1B). As little difference was evident in protoplasting efficiency between 8 h and 18 h, we chose the latter throughout our work for convenience. Protoplasts prepared as such showed spherical shape and were extremely sensitive to detergents such as SDS (Fig. 1C), indicating that protoplasting was successful.

We next compared regeneration efficiency of protoplasts on GK medium with various osmoprotectants, and it was evident that 0.4 M MgSO_4_ gave the best results (Fig. S2A). To test whether the prepared protoplasts were suitable for transformation, we first surveyed their sensitivity to several selection reagents and found that with 0.4 M MgSO_4_ as the osmoprotectant, only phosphinothricin (PPT, also known as glufosinate) inhibited growth, with G418 and hygromycin being virtually ineffective (Fig. S2B). Switching 0.4 M MgSO_4_ to 1 M sorbitol did sensitize protoplasts to G418 and hygromycin, but their respective resistant cassettes amplified from the pFA6a plasmids (59) did not confer sufficient resistance (data not shown). Therefore, we focused on PPT and constructed a plasmid expressing the *Streptomyces hygroscopicus bar* gene encoding for phosphinothricin *N*-acetyltransferase to confer PPT resistance, driven by the endogenous *gpd* promoter (Fig. 1D). The first two exons and the first intron of the *gpd* gene were retained as their presence was reported to increase the expression of heterologous genes (35). Protoplasts incubated with *Swa*I-linearized plasmid yielded PPT-resistant transformants on the GK medium, but not those without DNA (Fig. 1E). Subsequent molecular diagnosis showed successful amplification of the *bar* cassette in the transformant, but failure to amplify the *mnp1-bar* junction implied that integration was non-homologous (Fig. 1F).

### Loss of Ku70 promotes mononucleate conidiation in *P. chrysosporium*

**Fig 2:**
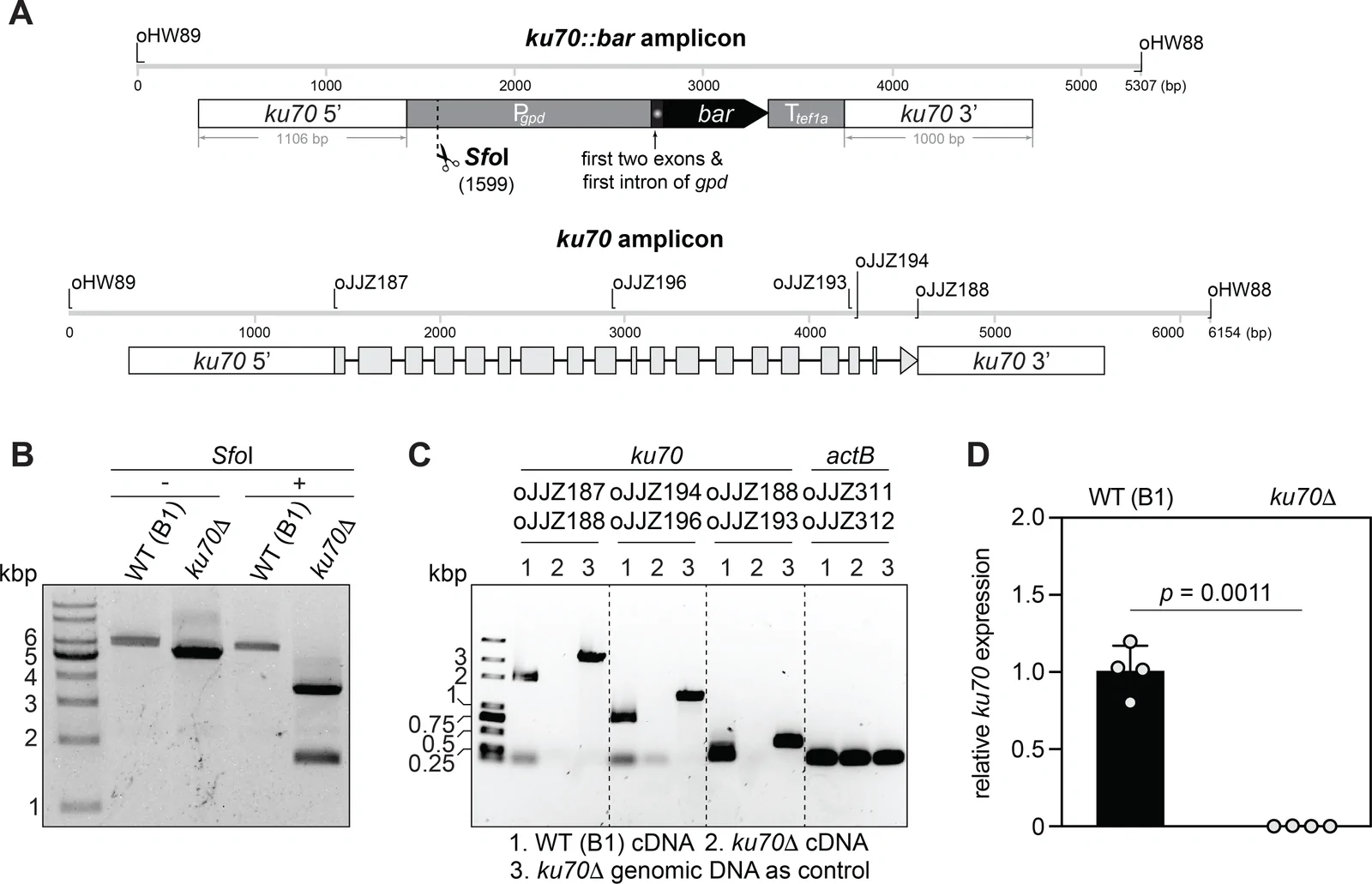
Construction and verification of a homozygous *ku70*Δ mutant. (**A**) Schematic of intact *ku70* and *ku70*Δ amplicons. Light gray boxes indicate predicted exons. Homologous sequences are shown as white boxes, i.e., *ku70* 5′ (1,106 bp) and 3′ (1,000 bp). Primers for diagnostic PCR and reverse transcription (RT) coupled with (quantitative) PCR are also denoted. (**B**) PCR diagnosis of WT and *ku70*Δ using oHW88 and oHW89 in the presence or absence of *Sfo*I. Characterization of WT and *ku70*Δ by RT-PCR (**C**) or RT-qPCR (**D**). Mean ± s.d. (*n* = 4 biological replicates) for RT-qPCR. The *P*-value was calculated using Welch’s *t* test in GraphPad. Amplicons from genomic DNA are typically longer due to the presence of introns, serving as controls for contamination of genomic DNA in cDNA preparation. oJJZ187/oJJZ188, oJJZ194/oJJZ196, and oJJZ188/oJJZ193 are different primer pairs to detect *ku70* cDNA, with oJJZ311/oJJZ312 for cDNA of the β-actin gene(s) (*actB*). oJJZ188 and oJJZ193 were used for RT-qPCR.

After reestablishing an effective transformation protocol, we decided to test whether loss of Ku70 might improve HR. Ku70 had not been investigated in *P. chrysosporium*, and a putative homologue (protein ID 6342975) was identified by Basic Local Alignment Search Tool (BLAST) using human Ku70 as bait. Conserved domain search (60) predicted it to be a Ku70 homologue (Fig. S3A), and for simplicity, we would refer to 6342975 as Ku70. However, this nomenclatural preference should not be interpreted as functional confirmation. We next constructed a linear DNA cassette bearing P*_gpd_*-driven *bar* sandwiched between the upstream and downstream sequences of *ku70* (Fig. 2A). The *ku70::bar* cassette was amplified from pHW10 using primers seHW9 and seHW14 (Fig. S3B) and transformed into homokaryotic B1 to knock out *ku70*. After screening approximately 140 transformants, we identified a putative homozygous *ku70*Δ mutant. Diagnostic PCR using primers flanking the *ku70* 5′ and 3′ sequences (oHW88 and oHW89 in Fig. 2A) showed that the WT amplicon, as expected, was slightly larger, which importantly was absent in the *ku70*Δ mutant (Fig. 2B). The *Sfo*I site in the *ku70::bar* amplicon (Fig. 2A) enabled us to use restriction digestion to further confirm that the *ku70*Δ mutant was homozygous (Fig. 2B). We also performed diagnostic PCR using oHW320 and oHW321, two primers specific for sequences on pHW10, just outside the *ku70* 3′ and 5′ regions of the *ku70::bar* cassette, respectively (Fig. S3B). If HR occurred, their priming sites would be lost due to crossovers at the homologous sequences, whereas non-homologous recombination (non-HR) would likely still allow them to anneal. Indeed, one transformant derived from non-HR gave an expected amplicon, which importantly was absent in *ku70*Δ (Fig. S3B), indicating that additional non-HR was unlikely in *ku70*Δ. Moreover, reverse transcription coupled with PCR showed that *ku70* expression was readily detectable in WT, but not in *ku70*Δ (Fig. 2C). Reverse transcription coupled with quantitative PCR (qPCR) further confirmed that there was little, if at all, *ku70* expression in *ku70*Δ (Fig. 2D). Taken together, these results indicate that our *ku70*Δ is a homozygous mutant with no evidence for additional non-HR.

**Fig 3:**
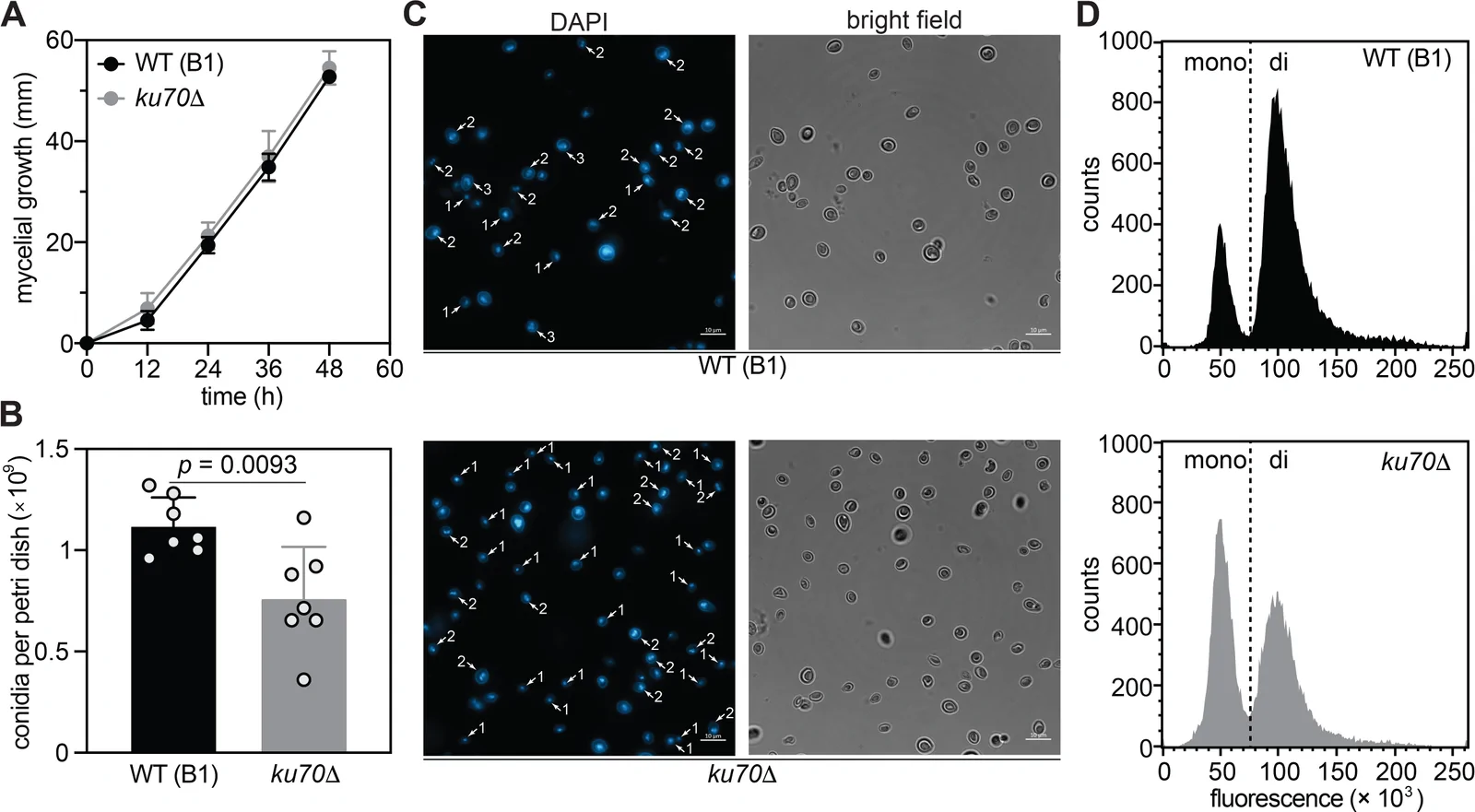
Loss of Ku70 promotes mononucleate conidiation. (**A**) Loss of Ku70 did not discernibly affect mycelial growth. Mean ± s.d. (*n* = 7 biological replicates). (**B**) Loss of Ku70 slightly lowered conidial yield. Mean ± s.d. (*n* = 7 biological replicates). The *P*-value was calculated using Welch’s *t* test in GraphPad. (**C**) Fluorescence microscopy showing that loss of Ku70 promoted mononucleate conidiation. Conidia were stained with DAPI, and the number of nuclei in each conidium, if determined confidently, is marked next to a white arrow. (**D**) FACS showing enrichment of mononucleate conidia in *ku70*Δ compared to WT. Conidia collected from GK plate were stained with propidium iodide and examined using a flow cytometer (see Materials and Methods for details).

Mycelial growth on GK plate was virtually indistinguishable between WT and *ku70*Δ (Fig. 3A; Fig. S3C), and the latter appeared to be slightly more sensitive to the DNA damaging reagent phleomycin (Fig. S3D). Loss of Ku70 slightly reduced conidial yield (Fig. 3B) but did not discernibly affect conidial morphology (Fig. 3C). Examination of conidia using fluorescence microscopy following 4’,6-diamidino-2-phenylindole (DAPI) staining revealed the prevalence of dinucleate conidia in WT, although mono- and tri-nucleate conidia were also visible (Fig. 3C), consistent with early reports (61–63). By comparison, loss of Ku70 significantly elevated the fraction of mononucleate conidia, while trinucleate conidia were hardly detectable (Fig. 3C). As microscopic inspection of the number of nuclei in conidia may not always be unequivocal, we next performed fluorescence-activated cell sorting (FACS) following propidium iodide staining and found that, in agreement with our microscopy results, the ratio between mono- and di-nucleate conidia was significantly increased in *ku70*Δ (Fig. 3D). Taken together, these data suggest that although homozygous loss of *ku70* minimally impacts mycelial growth of *P. chrysosporium*, Ku70 and, by extension, c-NHEJ play, directly or indirectly, an important role in regulating the number of nuclei during conidiation. This conclusion was further corroborated by subsequent complementation experiment after additional selection marker became available (see below).

### Loss of Ku70 significantly increases HR frequency

Early genetic studies in *P. chrysosporium* demonstrated that transformed DNA was often integrated randomly into the genome (33, 39), and there had been limited efforts to specifically target a gene(s) for deletion. Randall and Reddy attempted to inactivate the *lip2* gene via HR but to no avail since the transformed DNA was unexpectedly maintained as an episome (64). To test whether loss of Ku70 would increase HR frequency and improve genome editing precision, we chose as the target for proof of concept the *mnp1* gene encoding for Mnp1, one of the most thoroughly studied manganese peroxidases in *P. chrysosporium* (65–68).

To construct an *mnp1* deletion cassette, we would need a second selection marker in addition to the PPT-resistant *bar* gene that was used to replace *ku70*. Since neither hygromycin- nor G418-resistant cassette, as mentioned previously, driven by the same *gpd* promoter conferred measurable resistance, we resorted to isolation of uracil auxotrophs (ura^−^) in WT and *ku70*Δ using 5-fluoroorotic acid (5-FOA). To minimize fortuitous mutations, we avoided any mutagens such as UV and could readily obtain uracil auxotrophs in both strains. After phenotypic confirmation, e.g., failure to grow on GK minimal medium without uracil, we performed Sanger sequencing of *ura3* and *ura5*, encoding for orotidine-5′-phosphate decarboxylase and orotate phosphoribosyltransferase, respectively, two key enzymes in *de novo* pyrimidine synthesis, whose inactivation individually was sufficient to confer resistance to 5-FOA (69). Indeed, we identified mutations exclusively in *ura3* and *ura5*, and no phenotypic differences were discernible between them, irrespective of the presence or absence of *ku70*. Either the *ura3* or *ura5* mutant was successfully transformed to prototrophy (69), and without any preference, we randomly chose three isolates bearing *ura3* lesions for WT and *ku70*Δ for subsequent transformation studies (Table S4).

**Fig 4:**
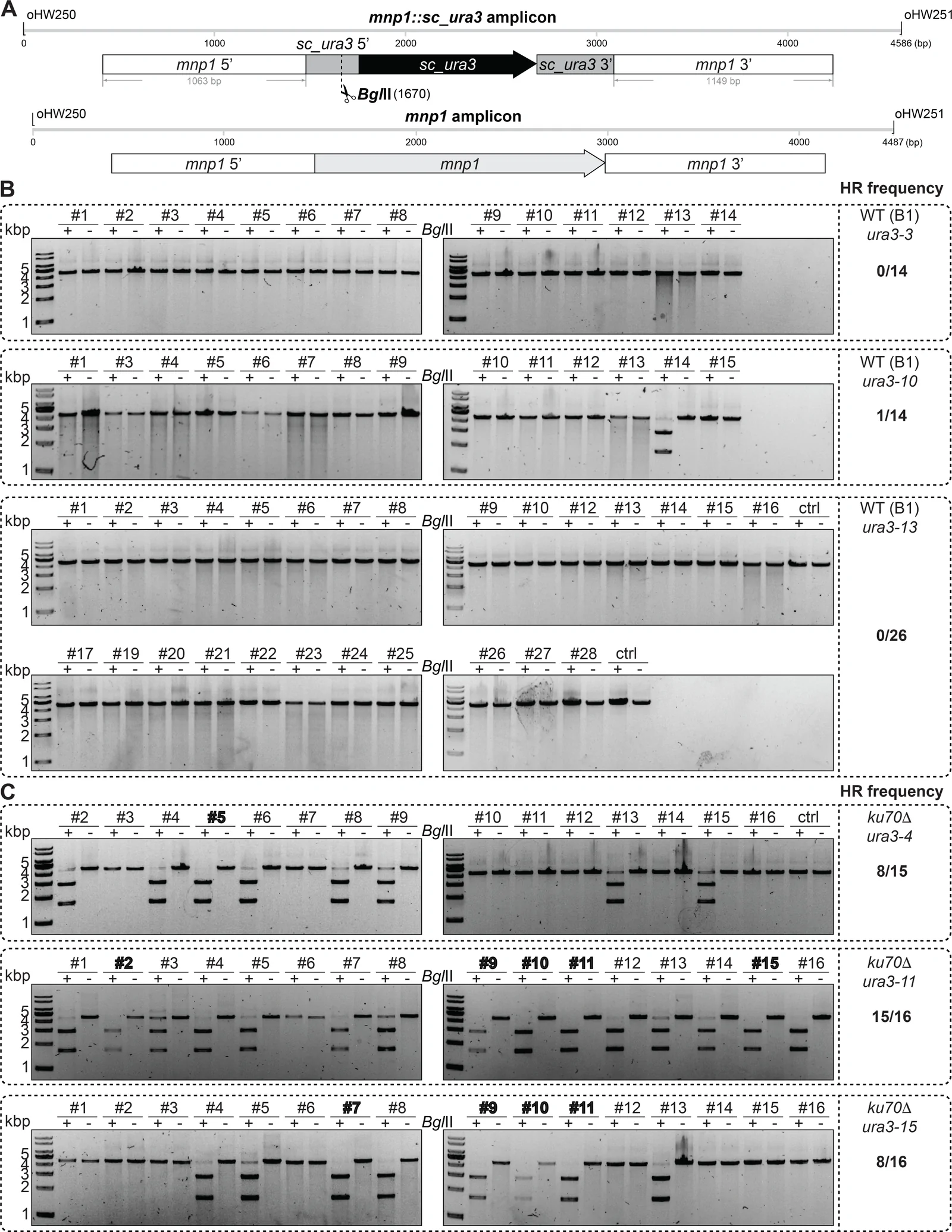
Loss of Ku70 significantly increases HR frequency. (**A**) Schematic of amplicons of intact *mnp1* and *mnp1*Δ. Homologous sequences are shown as white boxes, i.e., *mnp1* 5′ (1,063 bp) and 3′ (1,149 bp), with exons hidden for clarity. PCR diagnosis of transformants derived from WT ura^−^ (**B**) and *ku70*Δ ura^−^ (**C**) using primers oHW250 and oHW251 with or without *Bgl*II digestion. Information on the various *ura3* alleles (e.g., WT *ura3-3*) is available in Table S4, and putative homozygous *mnp1*Δ mutants are in boldface. Ctrl: amplicon of intact *mnp1*.

Linear DNA bearing *mnp1* 5′ and 3′ sequences, with *S. commune ura3* (*sc*_*ura3*) sandwiched in between, was amplified from pHW56 using seHW9 and seHW14 (Fig. S4A) and transformed into WT and *ku70*Δ ura^−^ strains. Prototrophic transformants were selected and confirmed on GK minimal medium. Genomic DNA was extracted and subjected to diagnostic PCR using primers oHW250 and oHW251, annealing to sequences flanking the 5′ and 3′ regions of *mnp1*, respectively (Fig. 4A). Since intact *mnp1* would give an amplicon of 4,487 bp and *mnp1*Δ 4,586 bp, it was difficult to differentiate between them simply based on size. This technical inconvenience, however, could be circumvented by restriction digestion with *Bgl*II, which would cut the *mnp1*Δ amplicon but spare the intact *mnp1* amplicon. Despite this demand for additional work, their similar amplicon sizes would be expected to minimize bias in amplification efficiency to allow for a more accurate estimation of the zygosity status (i.e., homozygous vs heterozygous) in the *mnp1*Δ mutants.

In WT, the three tested ura^−^ isolates showed very low HR frequency: we could identify only one heterozygous *mnp1*Δ in WT *ura3-10*, and all transformants derived from *ura3-3* and *ura3-13* contained intact *mnp1*, with a collective HR frequency at ~2% (1/54) (Fig. 4B). By contrast, transformants derived from the three *ku70*Δ ura^−^ isolates exhibited ≥50% HR frequency, with that of *ku70*Δ *ura3-11* even approaching greater than 90% (Fig. 4C). The collective HR frequency in *ku70*Δ was ~66% (31/47), significantly higher than that of WT. The observation that ten of the *ku70*Δ *mnp1*Δ double mutants appeared to be homozygous was noteworthy, accounting for ~32% of all HR transformants.

The transformants were subjected to additional tests. First, we used the aforementioned oHW320 and oHW321 to examine non-homologous integration of the *mnp1::sc_ura3* cassette (Fig. S4A). Like the *ku70::bar* cassette (Fig. S3B), homologous integration would prevent amplification due to the loss of their priming sites, whereas non-homologous integration would likely still retain their priming sites. Indeed, all non-HR transformants gave an amplicon of the expected size (~3.9 kb), whereas HR transformants yielded no amplification, except for *ku70*Δ *ura3-11* #3 (Fig. S4B and C). It showed clear evidence of HR at *mnp1* (Fig. 4C), and non-HR was indicated by the successful amplification with oHW320/oHW321 (Fig. S4C). There were two possible explanations: Both HR and non-HR occurred in a protoplast during transformation; the transformant was a mixture of two independent protoplasts, which might arise from protoplast fusion during transformation or failure to separate during selection. Currently, it was difficult to conclusively distinguish between these two possibilities. However, we favored the second explanation since transformation efficiency was generally very low in *P. chrysosporium*, and the probability of two independent events of DNA integration occurring simultaneously in a protoplast would be extremely low. Nonetheless, these results demonstrate that complementation using *S. commune ura3* is remarkably reliable and that HR and non-HR are largely mutually exclusive during transformation.

We also performed qPCR to estimate the copy number of the *mnp1::sc*_*ura3* cassette integrated into the *mnp1*Δ mutants. To this end, plasmid pJJZ81 was constructed bearing *mnp1::sc*_*ura3* and the putative *P. chrysosporium ku80* (Protein ID 6219778) at a 1:1 ratio (Fig. S5A) and used for establishing calibration curves. We found no compelling evidence for pervasive multi-integration of the *mnp1::sc_ura3* cassette in the *mnp1*Δ mutants since in most of the tested transformants, the ratio of *mnp1::sc*_*ura3* to the putative *ku80* was hovering below 1.5 (Fig. S5B). Although a few transformants did show a higher ratio than 1.5, e.g., *ku70*Δ *ura3-15* #5, none of them appeared to undergo additional non-homologous integration of the *mnp1::sc_ura3* cassette (Fig. S4C).

Taken together, these results demonstrate that loss of Ku70 significantly increases HR frequency in *P. chrysosporium* and that approximately one-third of the HR transformants are also homozygous. Moreover, our PCR and qPCR results (Fig. S4B, S4C, and S5B) support the notion that HR and non-HR are largely mutually exclusive during transformation of *P. chrysosporium*, with no clear evidence for prevalent multi-integration of the *mnp1::sc_ura3* cassette in the *ku70*Δ mutant.

### Ectopic expression of *ku70* suppresses mononucleate conidiation in *ku70*Δ

**Fig 5:**
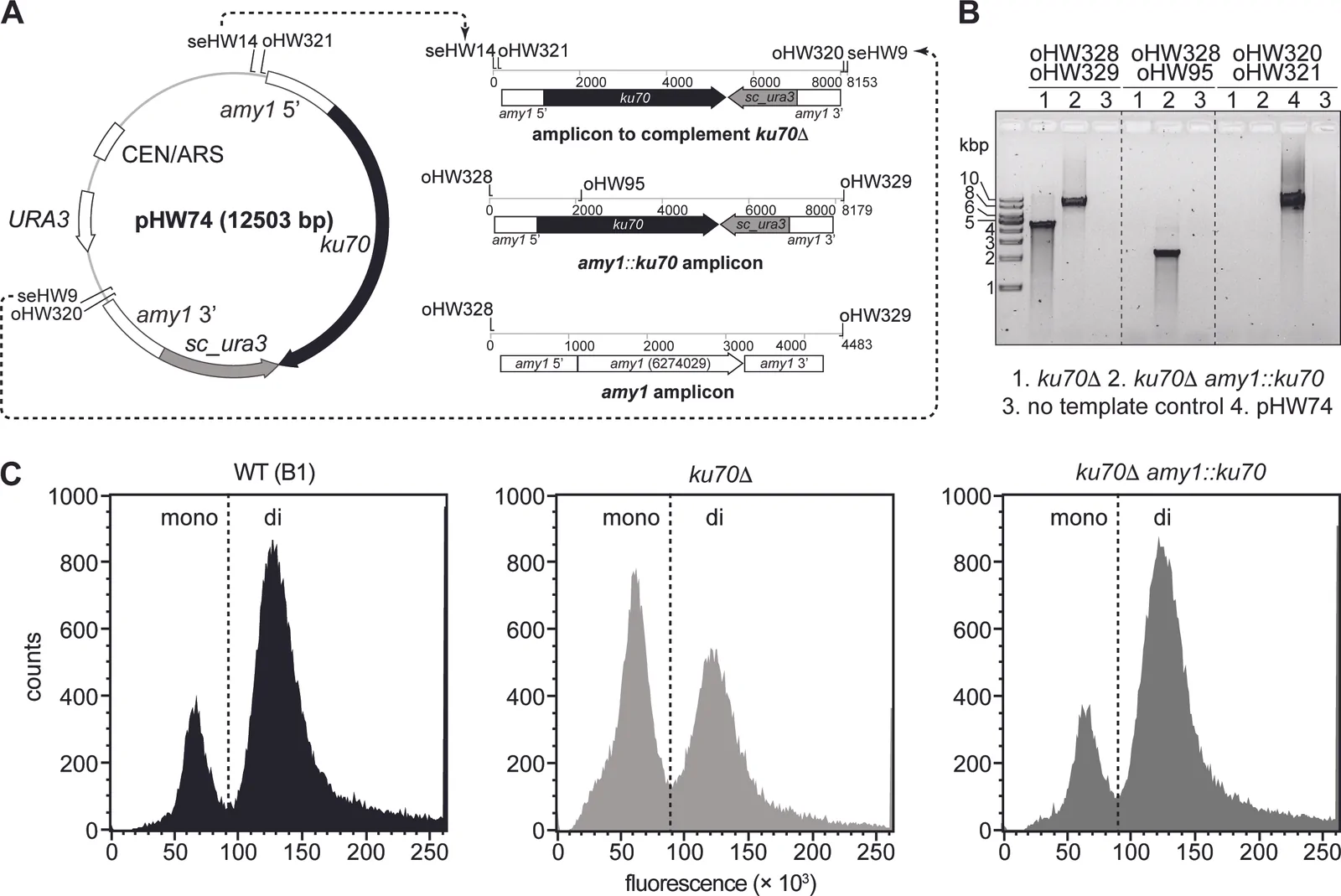
Ectopic expression of *ku70* suppresses mononucleate conidiation in *ku70*Δ. (**A**) Schematic of pHW74 used to amplify the *ku70* complementation cassette by seHW9 and seHW14, as well as the expected amplicons of *amy1::ku70* and intact *amy1* by oHW328 and oHW329. (**B**) Diagnostic PCR showing homozygous replacement of *amy1* with the *ku70* complementation cassette in the *ku70*Δ *amy1::ku70* mutant. (**C**) FACS showing suppression of mononucleate conidiation in *ku70*Δ by ectopically expressing *ku70*.

Lastly, the availability of *sc*_*ura3* as a second selection marker enabled us to further test the importance of Ku70 in conidiation. To this end, we sandwiched the endogenous *ku70*, including its putative promoter and terminator, as well as *sc*_*ura3*, between the 5′ and 3′ regions of a putative amylase gene (protein ID 6274029, referred to as *amy1*) (Fig. 5A). The *ku70* complementation cassette was amplified from pHW74 using seHW9 and seHW14 and transformed into *ku70*Δ *ura3-11* (Table S4). One putative homozygous *ku70*Δ *amy1::ku70* was isolated and further confirmed by diagnostic PCR with oHW328 and oHW329, two primers annealing just outside of the 5′ and 3′ regions of *amy1*, respectively. The *ku70*Δ *amy1::ku70* mutant showed the expected *amy1::ku70* amplicon (8,179 bp) but not the intact *amy1* amplicon (4,483 bp) (Fig. 5B). Additional tests using oHW95 and oHW328 further confirmed homologous integration of the *ku70* complementation cassette at *amy1*, and no evidence was found for non-homologous integration using oHW320 and oHW321 (Fig. 5B). Importantly, analysis using FACS revealed that the proportion of mononucleate conidia was significantly reduced in the homozygous *ku70*Δ *amy1::ku70* mutant to the extent that it was virtually indistinguishable from WT (B1) (Fig. 5C). Collectively, this complementation experiment provides further evidence that loss of Ku70 promotes mononucleate conidiation in *P. chrysosporium*.

## DISCUSSION

### Loss of Ku70 significantly improves genome editing precision in *P. chrysosporium*

*P. chrysosporium* is the best studied WRF with respect to its ligninolytic machinery and regulation (70). Genome editing with precision, e.g., in the case of gene knockout, is essential to elucidating the mechanisms of its ligninolytic actions and to constructing superior chassis for industrial application. Early efforts by the Gold group established the first transformation method based off auxotroph complementation (33), which was quickly adopted and expanded to include additional selection markers (34, 58, 64, 71). Despite their usefulness, these methods are generally plagued by low precision, a common problem in filamentous fungi, as the transformed DNA is rarely processed through HR. Therefore, significant efforts must be devoted to identifying a mutant of desired genotype, e.g., homozygous deletion of a gene of interest, to investigate its functional importance. Such endeavors are further impeded by the fact that conidia (61–63) or basidiospores (72, 73) of certain *P. chrysosporium* strains, common starting materials for protoplast-based transformation, are predominantly dinucleate. Although an RNAi-based approach was previously established to achieve more specific control in repressing the expression of certain genes, the knockdown efficiency at the mRNA level varied, ranging from 17% to 76% (37). Lastly, CRISPR-Cas9-based genome editing has found tremendous success in genetic engineering of filamentous fungi (74), but it has yet to be established for *P. chrysosporium* (75). Directed by guide RNA, the Cas9 endonuclease cleaves DNA with remarkable efficiency and precision, significantly accelerating genome editing efforts. Future studies are warranted to develop this powerful tool for *P. chrysosporium* to understand its ligninolysis and to build superior chassis for industrial applications.

In this work, to improve genome editing precision in *P. chrysosporium*, we first isolated meiotic homokaryons from the well-characterized heterokaryon BKMF-1767, followed by whole-genome sequencing to clarify their genetic background. We then reestablished protoplast-based transformation by replacing the discontinued Novozyme 234 with Lywallzyme and achieved excellent protoplasting efficiency. It should be noted that the combination of Yatalase (Takara) and Cellulase Onozuka R-10 (Wako) also worked effectively for protoplasting purposes (36), which together with Lywallzyme will certainly offer more options. Importantly, homozygous deletion of *ku70* to inactivate c-NHEJ, which has not been attempted in *P. chrysosporium*, significantly increased HR frequency at *mnp1* from ~2% to ~66% (Fig. 4B and C), while minimally impacting growth (Fig. 3A). Interestingly, approximately one-third of the *mnp1*Δ mutants appeared to be homozygous. As we have only tested homologous sequences up to 1 kb and the *mnp1* gene, it will be of interest to examine in future studies whether extending homology (e.g., 2 kb) would further improve HR frequency, as observed in *N. crassa* (42), and whether there might be variations in HR frequency at different genomic loci. Lastly, the observation that HR frequency failed to reach 100% in the homozygous *ku70*Δ mutant suggests that NHEJ is not completely abolished, and this might be due to the action of Ku-independent alternative end joining pathways (76, 77). Nevertheless, from a practical standpoint, if one assumes that genome-wide HR frequency does not vary significantly in the absence of c-NHEJ, our *ku70*Δ mutant should serve as a valuable tool to understand and harness the remarkable ligninolytic capabilities of *P. chrysosporium* since a minimum of 20 transformants would be sufficient to isolate three to four homozygous mutants.

### Loss of Ku70 promotes mononucleate conidiation in *P. chrysosporium*

Although the Ku proteins have been extensively investigated in filamentous fungi, a link between c-NHEJ and the number of nuclei in conidia, to our best knowledge, has not been reported. Strain background appears to influence the number of nuclei during conidiation of *P. chrysosporium* (61–63). For instance, while ME446 primarily yields mononucleate conidia, BKMF-1767, on the other hand, produces predominantly dinucleate conidia. Similar to its parental BKMF-1767, meiotic homokaryon B1 is also a proficient producer of dinucleate conidia (Fig. 3C, 3D, and 5C). Conidiation in *P. chrysosporium* is poorly understood at the molecular and cellular levels, and currently it remains elusive as to how loss of Ku70 could tilt the ratio between mononucleate and dinucleate conidia. If asymmetric mitosis is shared between *P. chrysosporium* and *Aspergillus* during conidiation (78), perhaps loss of Ku70 may dampen mitosis; therefore, fewer nuclei are available for packing into each conidium. Or perhaps, as in *Aspergillus oryzae,* where multinucleate conidia are formed by nuclear migration (79), loss of Ku70 may impede this process to promote mononucleate conidiation. Both speculations, certainly warranting future investigations, are nevertheless consistent with the mild conidiation defects observed in the *ku70*Δ mutant (Fig. 3B).

## MATERIALS AND METHODS

### Strains and growth conditions

*P. chrysosporium* BKMF-1767 (ATCC24725) (80) was purchased from China Center of Industrial Culture Collection (CICC) and maintained on potato dextrose agar (PDA). GK medium was prepared as described previously (35), which contained a mineral solution as specified by Kirk et al. (12), 1% glucose, 12 mM ammonium tartrate, and 10 mM potassium phosphate, at pH 5.0. MYV medium was prepared as described by Alic et al. (33), with the following modification: Ammonium nitrate was replaced by ammonium sulfate due to the latter’s easier access. Mycelial growth was measured as follows. A mycelial plug (~8 mm in diameter) from a 5-day-old culture on GK plate was mounted in the center of a fresh GK plate. Two diametral lines, perpendicular to each other, were drawn, and growth was estimated by averaging mycelial expansion from the perimeter of the plug along these two lines every 12 h.

### Plasmids and cloning

The *bar* gene encoding for *S. hygroscopicus* phosphinothricin *N*-acetyltransferase was codon-optimized for *P. chrysosporium* and synthesized by Genewiz (Suchow, China), which was subsequently cloned between the endogenous *gpd* promoter and *tef1a* terminator. Unless stated otherwise, all plasmids were derived from pKQL57 (Table S4), constructed using Gibson assembly, and verified by Sanger sequencing.

### Isolation of meiotic homokaryons

Conidia of *P. chrysosporium* BKMF-1767 were washed off PDA plates with sterile distilled water and filtered through degreased cotton. Fruiting was induced following the method of Gold and Cheng (56) with minor modifications. Briefly, conidia were plated on five-fold diluted Vogels medium N supplemented with 1% xylose. Plates were incubated with the lid down at 28°C under continuous lighting for 2 weeks. Basidiospores ejected onto the lid were collected with sterile distilled water and plated onto Gold B screening plate after proper dilution. Conidia were collected from mycelia derived from single basidiospores for further analysis.

### Whole-genome sequencing and data analysis

Libraries were prepared and sequenced by BIOZERON (Shanghai, China). Briefly, total DNA was extracted from *P. chrysosporium* mycelia using the EZNA DNA isolation kit (Omega Bio-Tek) following the manufacturer’s recommendations. Paired-end genomic DNA libraries, with an average insert size of ~450 bp, were constructed following Illumina’s recommendations and sequenced on the Illumina NovaSeq 6000 platform (150 bp ×2).

The *P. chrysosporium* RP-78 genome (version 4.0) was downloaded from Joint Genome Institute (JGI) and used as a reference for sequence alignment and comparison. Raw paired-end sequences were trimmed using Fastp (81) and aligned to the RP-78 genome using the BWA package. Aligned SAM files were coordinate-sorted and converted to BAM files, followed by identification of duplicate reads using the Picard package (http://broadinstitute.github.io/picard). BAM files were indexed and sorted by SAMtools (82). SNPs/indels were called, compared between B1 and B3, and annotated by GATK HaplotypeCaller, Mutect2, and ANNOVAR, respectively (83, 84). DELLY (85) was implemented to identify structural variations (SVs). Tentative differences between B1 or B3 and RP-78 are summarized in Tables S1 and S2, respectively. B1 and B3 were compared using GATK Mutect2 by reciprocally setting one as “normal” and the other as “tumor”, i.e., B1 as “normal” and B3 as “tumor”, and then B1 as “tumor” and B3 as “normal”. GATK Mutect2 reports differences between “tumor” and the reference (i.e., RP-78 genome) only if they are also absent from the matched “normal.” GATK FilterMutectCalls was employed to remove low-quality variant calls, and only entries bearing the “PASS” filter were reported (Table S3) and inspected further in IGV. Tentative SVs between B1 or B3 and RP-78 bearing the “PASS” FILTER were also examined in IGV, and no SVs could be confidently identified between B1 and B3.

### Preparation of protoplasts

*P. chrysosporium* was cultured at 37°C on PDA for 5 to 8 days. Conidia were collected in sterile distilled water and filtered through autoclaved degreased cotton. Approximately 2 × 10^8^ conidia were inoculated in 100 mL of modified MYV, supplemented with 0.1 g L^−1^ uracil if auxotrophic strains were used, and incubated at 37°C and 150 rpm for 4.5 h. Swollen conidia were collected by centrifugation and washed twice with OM Buffer (0.5 M MgSO_4_ and 50 mM maleic acid, at pH 5.9). Approximately 4–5 × 10^7^ swollen conidia were incubated in 1 mL of OM Buffer with 20 mg of Lywallzyme (Institute of Microbiology, Guangdong Academy of Sciences, China) at 30°C with gentle shaking for 18 h. Protoplasts were pelleted by centrifugation and washed twice with SM Buffer (1.0 M sorbitol and 20 mM MOPS, at pH 6.3), before being finally resuspended in SMC Buffer (1.0 M sorbitol, 50 mM CaCl_2_, and 20 mM MOPS, at pH 6.3).

### Transformation

*P. chrysosporium* protoplasts were transformed with either restriction-enzyme linearized plasmid or linear DNA amplified from the plasmid as previously described (35) with minor modifications. Briefly, 2 × 10^6^ protoplasts in SMC Buffer were mixed with 1 to 3 μg of linear DNA in a total volume of 160 μL and incubated on ice for 10 min. The protoplast-DNA mixture was carefully underlaid with 160 μL of 44% (wt/vol) PEG solution (PEG3350 in 50 mM CaCl_2_ and 10 mM MES, at pH 6.75), mixed gently after 10-min incubation on ice, followed by an additional 10-min incubation on ice. The protoplast-DNA-PEG solution was mixed with 320 μL of SMC Buffer, and after a brief incubation at 37°C for 5 min, the mixture was added to 15 mL of regeneration medium (GK with 0.4 M MgSO_4_ and 1% agar) supplemented with 200 μg mL^−1^ PPT and poured into Petri dishes, followed by incubation at 37°C. Transformants were further verified by their ability to grow on GK with PPT.

### RNA isolation and reverse transcription

*P. chrysosporium* mycelia were collected from GK plate, snap-frozen in liquid nitrogen, and stored at −80°C. Frozen samples were resuspended in AE Buffer (0.3 M NaOAc, pH 4.5, 10 mM EDTA) followed by addition of an equal volume of acidic phenol (pH 4.2). Cells were lysed using acid-washed glass beads on a bead beater (four cycles of 1-min beating followed by 1-min cooling on ice). Cell debris and glass beads were removed by centrifugation. The aqueous phase was transferred into a clean centrifuge tube and extracted with an equal volume of acidic phenol, followed by a third extraction with chloroform to remove the residual phenol. RNA was ethanol-precipitated, washed with 70% ethanol, dried, and dissolved in nuclease-free water. Total RNA was treated with DNase I, extracted once with phenol:chloroform (1:1), and precipitated with ethanol. Purified RNA was examined electrophoretically and spectrophotometrically, and cDNA was synthesized using the ReverTra Ace qPCR RT Master Mix (Toyobo).

### qPCR

qPCR was performed on the Applied Biosystems QuantStudio 1 Real-Time System (Thermo). A typical reaction in 10 µL contained 5 ng of cDNA, 0.2 µM primers, and 5 µL of SupRealQ Ultra Hunter SYBR qPCR Master Mix (Vazyme). The β-actin gene(s) served as an internal standard using primers published previously (86). Relative gene expression was estimated using the 2^-ΔΔCt^ method (87), and the data represented four biological replicates, with four technical replicates for each. See Table 1 for primer sequences.

**TABLE 1:** List of primers

Primer	Sequence (5′ to 3′)	Purpose	Size	Ref
oHW1	CGAAACACTCAGTCAACGTG	To amplify the *gpd* promoter and fuse with *bar*	1,358 bp	This work
oHW32	CTTTGACCTGGAAAGCGAAGTCAGCACGCGGTCGTCGCGGTC GGG CTGTGTGGTGTACTGACCGGCATGTTCAAGTAGTGTAGGGGTG			
oHW35	GTGCTGACTTCGCTTTCCAGGTCAAAGCAA GCCCAGAACGACGCCCGGC	To amplify *bar* and fuse with the *gpd* promoter	582 bp	This work
oHW36	CAGTCTAAATCTCGGTGACG			
oHW37	CTGCCCGTCACCGAGATTTAGACTGCTTAGATGTATTCTC	To amplify the *tef1a* terminator and fuse with *bar*	422 bp	This work
oHW6	GCAGGGCCTTTATGGCACCTG			
oHW19	TTTTACAACGTCGTGACTG	To amplify the vector backbone from pKQL57 for pHW8	4,726 bp	This work
oHW20	GAGGAGAGAAGTGTAGCCTGTTCACAGCGCGCTTGGCGTAATCATG			
oHW7	GTGCCATAAAGGCCCTGCGCCTCTATCCACATGCGGTATC	To amplify the *mnp1* 3’ region and fuse with the *bar* cassette	1,192 bp	This work
oHW21	TTTTCCCAGTCACGACGTTGTAAAAAAGGAGTGCCAGCGCTAC			
oHW9	GTGAACAGGCTACACTTCTC	To amplify the *mnp1* 5’ region and fuse with the *bar* cassette	1,080 bp	This work
oHW10	CGTTGACTGAGTGTTTCGCTGGGATACCAAGCCGTTGAC			
oHW52	GCCGAGGGCGATAACATATTTG	To amplify the *ku70* 5’ region and fuse with the *bar* cassette	1,132 bp	This work
oHW53	GACCTGCACGTTGACTGAGTGTTTCGTTTGCCTGGGGTGGTGGTCG			
oHW55	CATAAAGGCCCTGCTGCAGGTCAACCACGATCTC	To amplify the *ku70* 3’ region and fuse with the *bar* cassette	1,014 bp	This work
oHW56	CTGTGAGATCAAGAACAAGG			
oHW57	CAACCTCCTTGTTCTTGATCTCACAGTTTTACAACGTCGTGACTG	To amplify the vector backbone from pKQL57 for pHW10	4,751 bp	This work
oHW58	CGACAAATATGTTATCGCCCTCGGCGCGCGCTTGGCGTAATCATG			
oHW88	GATCATCTCCTGCACGATGTC	To examine *ku70* deletion	5,307 bp for *ku70*Δ; 6,154 bp for intact *ku70*	This work
oHW89	GCTGCACCACAACTCGTTTG			
oHW235	GCCTCTATCCACATGCGGTATC	To amplify the vector backbone from pHW8 and fuse with *sc_ura3*	6,912 bp	This work
oHW238	CTGGGATACCAAGCCGTTGAC			
oHW236	GTGAGATACCGCATGTGGATAGAGGCG GATCCGACGTGGAGGAGCCG	To amplify *sc_ura3*	1,665 bp	This work
oHW237	TCCGGTCAACGGCTTGGTATCCCAGTCCA GCTCGACCTTGCGCCGCTTG			
oHW250	CAGAATGTCTTACGTTCACCCAGAC	To examine *mnp1* deletion	4,586 bp for *mnp1*Δ; 4,487 bp for intact *mnp1*	This work
oHW251	GACCTCTTGTACGCAACGTTGACAG			
oHW320	GCTGCAAGGCGATTAAGTTG	To detect nonhomologous integration of amplicons amplified from pHW10, pHW56, and pHW74 using seHW9 and seHW14	4,515 bp for pHW10; 3,925 bp for pHW56; 7,902 bp for pHW74	This work
oHW321	CACACAGGAAACAGCTATGACC			
seHW14	GGCCGATTCATTAATGCAG	To linearize the plasmid for transformation	4,766 bp for pHW10; 4,176 bp for pHW56; 8,153 bp for pHW74	This work
seHW9	CATTCGCCATTCAGGCTG			
*lipI* F	TATATGCCTCTGAGCTCCTG	To distinguish between homokaryons and heterokaryons	2,229 bp and 479 bp for the heterokaryon; 2,229 bp for the homokaryon	(47)
*lipI* R	ACGTCTGTCTAGAAGTATGC			
oJJZ187	ATGGCCTCCTACGACGAATGG	RT-PCR primers for *ku70*	3,157 bp for genomic DNA; 1,968 bp for cDNA	This work
oJJZ188	TCAGGAGTGGCCATCTAACC			
oJJZ188	TCAGGAGTGGCCATCTAACC	RT-PCR and RT-qPCR primers for *ku70*	376 bp for genomic DNA; 180 bp for cDNA	This work
oJJZ193	GACCCAGGCGAGCCTAGC			
oJJZ194	CCTACGCACTTCCTCCTC	RT-PCR primers for *ku70*	1,322 bp for genomic DNA; 766 bp for cNDA	This work
oJJZ196	CGTGACCAGGGTGACTGC			
oJJZ311	ACTCTGGTGATGGTGTCTC	RT-PCR and RT-qPCR primers for β-actin gene(s)	149 bp	(86)
oJJZ312	TGTGGTGTTGAAGGGGTAA			
oJJZ414	CAAAGAACAAAGCTTGCC	To detect *sc_ura3* using qPCR	81 bp	This work
oJJZ415	GAGCTTATGAGCGGCGGT			
oJJZ428	CAAGTCGAAGGCAAAGCTGCG	To detect putative *ku80* (protein ID 6219778) using qPCR	148 bp	This work
oJJZ429	GAAGTCGTCTAGGGGGTGCAG			
oHW328	CTCCGACGGATGCAAACAGAC	To examine *ku70* complementation at putative *amy1* (protein ID 6274029)	8,179 bp for *amy1::ku70*; 4,483 bp for intact *amy1*	This work
oHW329	CGAGAGAGAAACGGACGATC			
oHW328	CTCCGACGGATGCAAACAGAC	To examine *ku70* complementation at *amy1*	2,149 bp for *amy1::ku70*	This work
oHW95	GTCTGTGACGAGGAACACTC			
oHW19	TTTTACAACGTCGTGACTG	To amplify the vector backbone from pHW56 for pHW74	4,700 bp	This work
oHW273	GCGCGCTTGCGTAATCATG			
oHW291	ATGACCATGATTACGCCAAGCGCGCCATCACATGAGAGAGGAAGTC	To amplify *amy1* 5’ and fuse with *ku70*	1,024 bp	This work
oHW362	CGAACTACACCGTCCCTAGCTACATGGTGCTGTGCTCTGGCGCAG			
oHW364	ATGTAGCTAGGGACGGTGTA	To amplify *ku70* and fuse with *amy1* 5’	4,215 bp	This work
oHW365	GACGGCTGTACGCTTTGCAG			
oHW272	TCCAGCTCGACCTTGCGCCG	To amplify *sc_ura3* from pHW56 and fuse with *amy1* 3’	1,639 bp	This work
oHW363	GCCTACTGCAAAGCGTACAGCCGTCG GATCCGACGTGGAGGAGCC			
oHW293	CCAAGCGGCGCAAGGTCGAGCTG GACAGTAGTACGATTTGCATC	To amplify *amy1* 3’ and fuse with *sc_ura3*	1,050 bp	This work
oHW294	TTTTCCCAGTCACGACGTTGTAAAA CACAAAACTATCGGACATGG			
oHW399	GAAGCAACAGAGCACCGAAC	To amplify the Scaffold 6 region	1,510 bp	This work
oHW400	CACGAGCATCTCTGTGGAAC			
oJJZ419	GAAAAGGACCCAGGTGGCAC	To amplify the vector backbone from pHW56 for pJJZ81	7,978 bp	This work
oJJZ420	GGCGTATCACGAGGCCCTTTC			
oJJZ423	GACGAAAGGGCCTCGTGATACGCCAT GACGGAACGAGCGGGG	To amplify *ku80*	3,185 bp	This work
oJJZ424	GAAAAGTGCCACCTGGGTCCTTTTCT CACTCGGTCATGCCGGTG			

To quantify *mnp1::sc_ura3* integrated into the *mnp1*Δ mutants, we constructed pJJZ81, bearing *mnp1::sc_ura3* and the putative *P. chrysosporium ku80*, at a 1:1 ratio (Fig. S5A). pJJZ81 was linearized by *Kpn*I, serially diluted, and used as a standard for qPCR. oJJZ414/415 and oJJZ428/429 were designed to detect *mnp1::sc_ura3* and putative *ku80*, respectively (Table 1), with the annealing temperature set for the former at 55°C and for the latter at 60°C. C_t_ mean and log_10_-transformed gene copy number were linearly correlated and used to extrapolate the corresponding gene copy number in a sample. Linearized pJJZ81 was always run alongside samples. At least three technical replicates were performed, and the arithmetic mean of their C_t_ values was used to estimate copy number.

### Isolation of uracil auxotrophs

Uracil auxotrophs were isolated as described previously (69) in the absence of mutagens. Approximately 2 × 10^7^ conidia were plated on GK plate supplemented with 0.7 g L^−1^ 5-FOA and 0.1 g L^−1^ uracil. The 5-FOA-resistant colonies were transferred onto GK plate with and without uracil to distinguish between homozygous and heterozygous mutants. The *ura3* and *ura5* genes of homozygous mutants were PCR-amplified and sequenced.

### Examination of conidia using fluorescence microscopy and FACS

Conidia were first revived from glycerol stock on GK or GK + 200 µg mL^−1^ PPT. After 5 days of incubation at 37°C, conidia were collected in sterile distilled water, and approximately 1 × 10^7^ conidia were seeded onto GK and incubated at 37°C for 5 days. Conidia were collected and prepared for either fluorescence microscopy or FACS as described below.

Microscopic analysis of conidia was performed as described previously (73). Briefly, approximately 1 × 10^7^ conidia were collected from GK plate, washed with sterile distilled water, and fixed in an ethanol-acetic acid solution (2:1) at room temperature for 10 min. Fixed conidia were pelleted and stained in the dark with 5 μg mL^−1^ DAPI for 1 min and 1.5 μg mL^−1^ DAPI for an additional 10 min. Stained conidia were pelleted, incubated in n-propanol for 10 min, pelleted again, resuspended in 20% glycerol, and examined under a Zeiss fluorescence microscope (Axio Imager.M2).

FACS was performed as described previously (88). Approximately 1 × 10^7^ conidia were collected from GK plate, washed with sterile distilled water, and incubated in 1 mL of Carnoy’s solution (ethanol:chloroform:acetic acid at a ratio of 6:3:1) with shaking at room temperature for 80 min. Conidia were pelleted, resuspended in 1 mL of absolute ethanol, and incubated with shaking at room temperature for 15 min. The fixed conidia were pelleted and gradually rehydrated by sequentially adding sterile distilled water in the volumes of 40 µL, 40 µL, 200 µL, 200 µL, and 200 µL. The rehydrated conidia were pelleted and washed twice with sterile distilled water, followed by treatment with 1.6 mg mL^−1^ RNase A at 50°C for 1 h and with 1.8 mg mL^−1^ proteinase K at 50°C for an additional hour. The treated conidia were pelleted, resuspended in ice-cold PBS, and stained overnight with 10 µg mL^−1^ propidium iodide in the dark at 4°C. The stained conidia were washed with PBS and re-stained with 2.5 µg mL^−1^ propidium iodide in the dark for 15 min. Alternatively, the RNase A- and proteinase K-treated conidia were stained with 10 µg mL^−1^ propidium iodide in the dark at room temperature for 1 h. Analysis of stained conidia was performed on LSRFortessa X-20 flow cytometer (BD), and data were processed using FlowJo (version 10.8.1).

## Statistical analyses

The *P*-values were calculated using Welch’s *t* test without assuming equal variances in GraphPad (version 10.4.1).

## Data Availability

Compressed raw sequencing files have been deposited in the NCBI Sequence Read Archive (SRA) under accession number PRJNA1422467.

## SUPPLEMENTAL MATERIAL

Figure S1 aem.00497-26-s0001.tif Characterization of meiotic homokaryons derived from BKMF-1767.

Figure S2 aem.00497-26-s0002.tif Characterization of *P. chrysosporium* protoplasts derived from B1.

Figure S3 aem.00497-26-s0003.tif Characterization of the *ku70* deletion mutant.

Figure S4 aem.00497-26-s0004.tif Characterization of transformants derived from WT and the *ku70* deletion mutant.

Figure S5 aem.00497-26-s0005.tif Quantification of *mnp1::sc_ura3* integrated into transformants by qPCR.

Table S1 aem.00497-26-s0006.xlsx Tentative SNPs, indels, and structural variations between B1 and RP-78.

Table S2 aem.00497-26-s0007.xlsx Tentative SNPs, indels, and structural variations between B3 and RP-78.

Table S3 aem.00497-26-s0008.xlsx Tentative SNPs and indels between B1 and B3.

Table S4 aem.00497-26-s0009.xlsx Strains and plasmids.

ASM does not own the copyrights to Supplemental Material that may be linked to, or accessed through, an article. The authors have granted ASM a non-exclusive, world-wide license to publish the Supplemental Material files. Please contact the corresponding author directly for reuse.
